# Effect of public health interventions during the first epidemic wave of COVID-19 in Cyprus: a modelling study

**DOI:** 10.1186/s12889-021-11945-9

**Published:** 2021-10-20

**Authors:** Ilias Gountas, Annalisa Quattrocchi, Ioannis Mamais, Constantinos Tsioutis, Eirini Christaki, Konstantinos Fokianos, Georgios Nikolopoulos

**Affiliations:** 1 Medical School, University of Cyprus, Palaios dromos Lefkosias Lemesou No.215/6, P.O.Box 20537, Nicosia, Cyprus; 2 Department of Primary Care and Population Health, University of Nicosia Medical School, Nicosia, Cyprus; 3 School of Sciences, European University, Nicosia, Cyprus; 4 School of Medicine, European University, Nicosia, Cyprus; 5 Department of Mathematics and Statistics, University of Cyprus, Nicosia, Cyprus

**Keywords:** Health policy, Mathematical modelling, COVID-19, Social distancing measures efficacy, Public health

## Abstract

**Background:**

Cyprus addressed the first wave of SARS CoV-2 (COVID-19) by implementing non-pharmaceutical interventions (NPIs). The aims of this study were: a) to estimate epidemiological parameters of this wave including infection attack ratio, infection fatality ratio, and case ascertainment ratio, b) to assess the impact of public health interventions and examine what would have happened if those interventions had not been implemented.

**Methods:**

A dynamic, stochastic, individual-based Susceptible-Exposed-Infected-Recovered (SEIR) model was developed to simulate COVID-19 transmission and progression in the population of the Republic of Cyprus. The model was fitted to the observed trends in COVID-19 deaths and intensive care unit (ICU) bed use.

**Results:**

By May 8th, 2020, the infection attack ratio was 0.31% (95% Credible Interval [CrI]: 0.15, 0.54%), the infection fatality ratio was 0.71% (95% CrI: 0.44, 1.61%), and the case ascertainment ratio was 33.2% (95% CrI: 19.7, 68.7%). If Cyprus had not implemented any public health measure, the healthcare system would have been overwhelmed by April 14th. The interventions averted 715 (95% CrI: 339, 1235) deaths. If Cyprus had only increased ICU beds, without any social distancing measure, the healthcare system would have been overwhelmed by April 19th.

**Conclusions:**

The decision of the Cypriot authorities to launch early NPIs limited the burden of the first wave of COVID-19. The findings of these analyses could help address the next waves of COVID-19 in Cyprus and other similar settings.

## Introduction

On January 30th 2020, the World Health Organization declared the outbreak of 2019 Coronavirus disease (COVID-19) a Public Health Emergency of International Concern [1], and on March 11th, 2020 a pandemic. The threat of COVID-19 to global health comes from the high percentage of the population that is not immune to the new pathogen SARS-CoV-2, the high transmissibility of SARS-CoV-2 in comparison to other respiratory viruses, and the potential substantial burden on healthcare systems [2, 3]. In the absence of effective therapeutics or vaccines, the majority of countries responded to COVID-19 with non-pharmaceutical interventions (NPIs) including social distancing measures, in order to limit viral spread, to ensure that the healthcare system would not be overwhelmed, and to prevent mass casualties.

The first COVID-19 cases in the Republic of Cyprus (~ 876,000 residents in the government-controlled area) were detected on March 9th, 2020, followed by a surge in diagnoses that peaked in late March to early April 2020. The response to the outbreak included several timely social distancing measures. More specifically, on March 10th schools and universities closed and, five days later, the access to the country was restricted and entertainment areas closed. On March 24th, most retail services closed and on March 31st social gatherings were prohibited [4]. A key public health response was also the wide test-trace-isolate strategy. During the first epidemic wave (by May 8th 2020), the testing rate in the Republic of Cyprus was 8932 per 100,000 population, which was significantly higher than in other European countries (e.g., the testing rate was 3018, 1217 and 926 per 100,000 population for the United Kingdom, the Netherlands, and Greece, respectively over the same period [4]. Finally, in order to meet the healthcare challenges anticipated by COVID-19, the number of COVID-19-designated intensive care unit (ICU) beds increased from 27 at the beginning of the outbreak to more than 50 by May 8th [5]. By May 8th, 21 COVID-19-related deaths (2.28 deaths per 100,000 population) were reported [4, 5].

During the COVID-19 pandemic, public health policymakers have to deal with a significant amount of data to make the optimal decision. Mathematical modeling is a useful tool that helps to synthesize information from different sources into a consistent framework that allows the examination of complex problems. It can provide important insights regarding the effectiveness of an intervention and inform pandemic planning policies. Although Cyprus’ response seems successful in controlling the first wave of COVID-19, it is important to quantitatively assess the effectiveness of the applied interventions. Notably, a significant number of countries have based their public health decisions on the results of these studies [3, 6–13].

In this work, we report the results of a dynamic COVID-19 modeling approach. Our aims were: a) to provide epidemiological estimates for the first wave of COVID-19 in the Republic of Cyprus such as infection attack ratio (IAR), infection fatality ratio (IFR), and case ascertainment ratio (CAR), b) to estimate the impact of NPIs, and examine possible outcomes in the hypothetical scenario where these measures were not implemented.

## Methods

### Description of the mathematical model

To simulate SARS-CoV-2 transmission and progression, a discrete time, stochastic, individual-based model, which categorizes the population into susceptible, exposed, infectious, or recovered (SEIR) individuals, was developed in programming language C++ (Dev-C++ v.5.11) (Supplementary, page 2, Fig. S1).

Every day, susceptible individuals might contact infected people and enter the exposed disease state before they become infectious. Infectiousness may start at least 1 to 2 days before the onset of symptoms [2, 14, 15]. Asymptomatic patients or patients with mild symptoms could recover from the disease after 5–7 days [16, 17]. Patients with severe or critical disease may need to enter a healthcare facility.

According to COVID-19 surveillance data in the Republic of Cyprus from March 1st to May 8th, 2020, the median time from symptom onset to hospital admission of patients with severe symptoms (i.e., requiring hospitalization) was 6.5 days (Interquartile Range (IQR): 4.5–9.5 days) (Table 1). The median time from symptom onset to hospital admission for patients who were later admitted to ICU was 5.5 days (IQR: 3.5–10 days). The overall median length of stay in ICU was 13.5 days (IQR: 8–28 days) [4, 5]. Cases in the ICU would either die or recover after spending a period of recovery time in a hospital bed. The probability of dying in ICU during the first wave was estimated at 41% [4, 20]. During the first epidemic wave in Cyprus, 891 cases were reported, 173 people received hospital care, and 32 cases have been admitted to ICU. Further details about the description of the model and the calibration procedure are available in the supplementary material. All healthcare inputs of the model were retrieved from publicly available data [4].

**Table 1:** Model parameters, assumptions, and references

Parameters	Value	References
**Cyprus population**	876,000	[18]
**Incubation period**	5 days	[19]
**Latent period**^**+**^	4 days (e.g., 1 day prior to the occurrence of symptom)	[14]
**Duration of infectious period until recovery**	6 days	[2]
**Duration from the development of symptoms to recovery**	5 days	[2]
**Median length of stay in Intensive Care Unit (ICU) bed**	13.5 days	[4]
**Disease mortality in ICU**	41%	[4, 20]
**Proportion of infections that will be either asymptomatic or mild (Fm)** **Proportion of infections that will progress to severe disease (Fs)** **Proportion of infections that will progress to critical disease (Fc)**	F_m_ = 86.0% F_s_ = 11.5% F_c_ = 2.5%	Fit to the observed deaths, hospitalized cases, and use of ICU beds
**Duration of hospitalization** **Duration of ICU and non-ICU stay**	15 days 20 days	[4]

### Model parameterization and examined scenarios

The validity of estimates from modeling studies depends on the quality of the data to which models are calibrated [3, 7, 21]. Under-ascertainment of COVID-19 cases has been recognized as a significant issue in many countries, since clinically severe cases are more likely to be recognized and tested [22]. Thus, our model was calibrated to match the trajectories of COVID-19-related deaths, hospitalized cases, and ICU bed use, as they are more reliable compared to data derived from diagnosed cases. Specifically, transmission rate, proportion of patients who would need hospital care or ICU, and effect of NPIs were varied until the model reproduced the observed trajectories. The end date was set to be May 8th, 2020, as this day was the end of the week in which lockdown restrictions were eased. The IAR was estimated using as numerator the number of total infections obtained from the model by May 8th and as denominator the total population of the Republic of Cyprus. The IFR was estimated using as numerator the number of reported COVID-19 related deaths and as denominator the number of total infections obtained from the model by May 8th. Finally, the CAR was estimated using as numerator the reported cases by May 8th and as denominator the number of total infections obtained from the model.

For each scenario, 1000 runs were performed. The 2.5 and 97.5 percentiles were also shown to include the appropriate uncertainty (stochastic variability). To measure the accuracy of the model’s prediction against the observed data, the least square method was used (i.e., minimize the square distance between observed and modeled data). Further details regarding the calibration procedure are available in the supplementary material.

### Examined scenarios

A ‘status quo’ scenario was used to generate predictions regarding the observed course of the outbreak. Additionally, two more scenarios were considered. Specifically, we implemented: 1) a hypothetical counterfactual scenario, wherein no interventions were implemented (neither social distancing measures nor increase in ICU bed capacity), to estimate how the outbreak would have unfolded if no interventions had taken place and 2) a scenario wherein only the number of ICU beds had increased (ICU-only scenario).

## Results

### Model fit

Estimates regarding model’s fit are presented in the supplementary document. Model’s status quo scenario accurately captures the observed trends in hospitalized cases, ICU bed use, and COVID-19-related deaths (Supplementary, page 6, Fig. S3-S5).

### The course of the first wave in Cyprus (march 1st - may 8th, 2020)

**Fig. 1:**
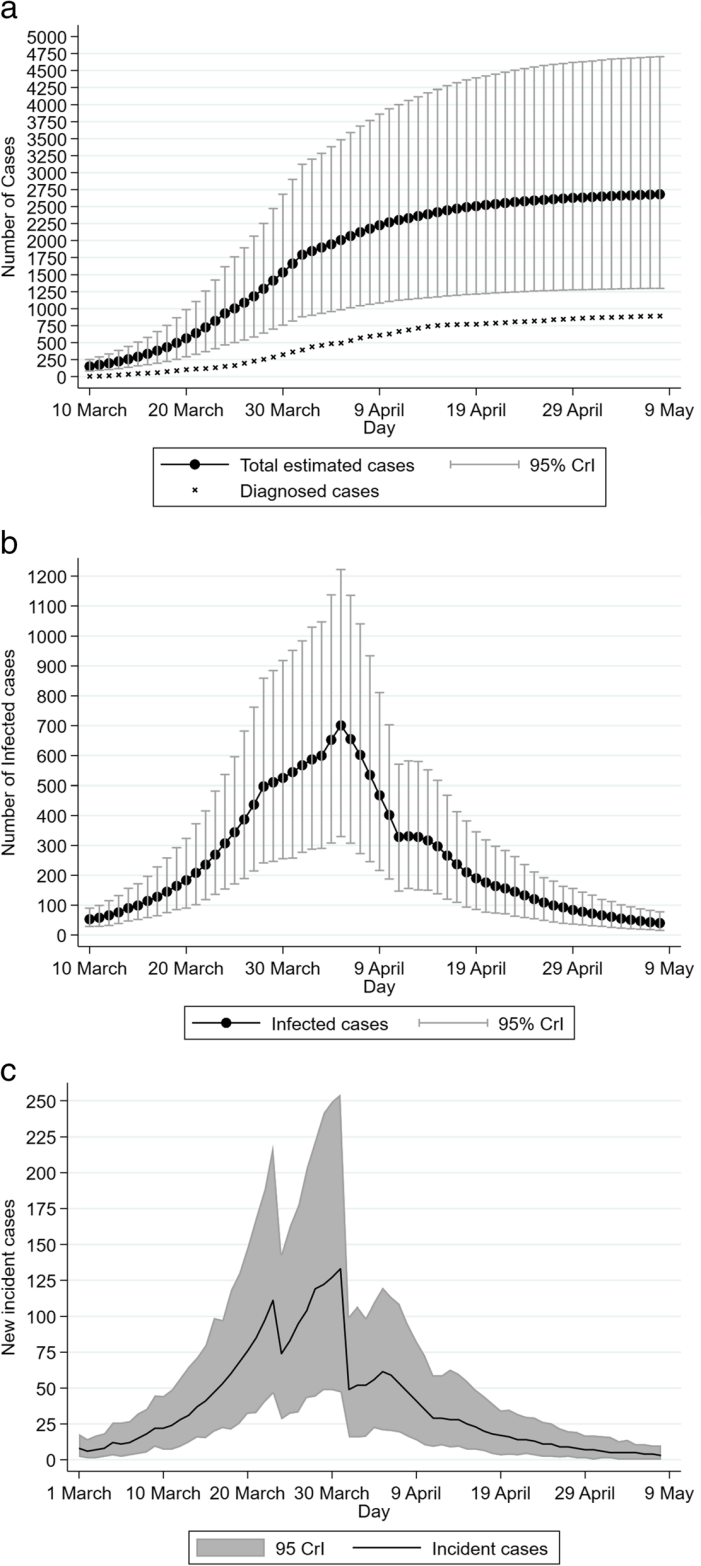
Model’s estimation regarding the first wave of COVID-19 in the Republic of Cyprus (1 March-8 May 2020): (A) Estimated total number of cases and diagnosed cases, (B) Estimated number of infectious individuals, (C) COVID-19 incident cases. The error bars show the 95% credible intervals (95% CrI) for the model projections

According to the model, the basic reproduction number (R_0_) was estimated at 2.66. The total estimated number of COVID-19 cases in the Republic of Cyprus by May 8th was 2690 (95% Credible Intervals (CrI): 1300, 4700) (Fig. 1a). Using as numerator the number of total infections obtained from the model, the IAR of the first epidemic wave was 0.31% (95% CrI: 0.15, 0.54%). Additionally, the CAR and the IFR of the first epidemic wave were 33.2% (95% CrI: 19.7, 68.7%) and 0.71% (95% CrI: 0.44, 1.61%), respectively.

At the end of the study’s prediction date (i.e., May 8th, 2020), it is estimated that there were 3 new infections per day (95% CrI: 0, 7) and 39 infectious cases (95% CrI: 15, 70). The model estimated that the number of new infections and the number of infected individuals peaked on March 31st and April 5th, 2020, respectively (Fig. 1b and Fig. 1c).

### Counterfactual scenario

**Fig. 2:**
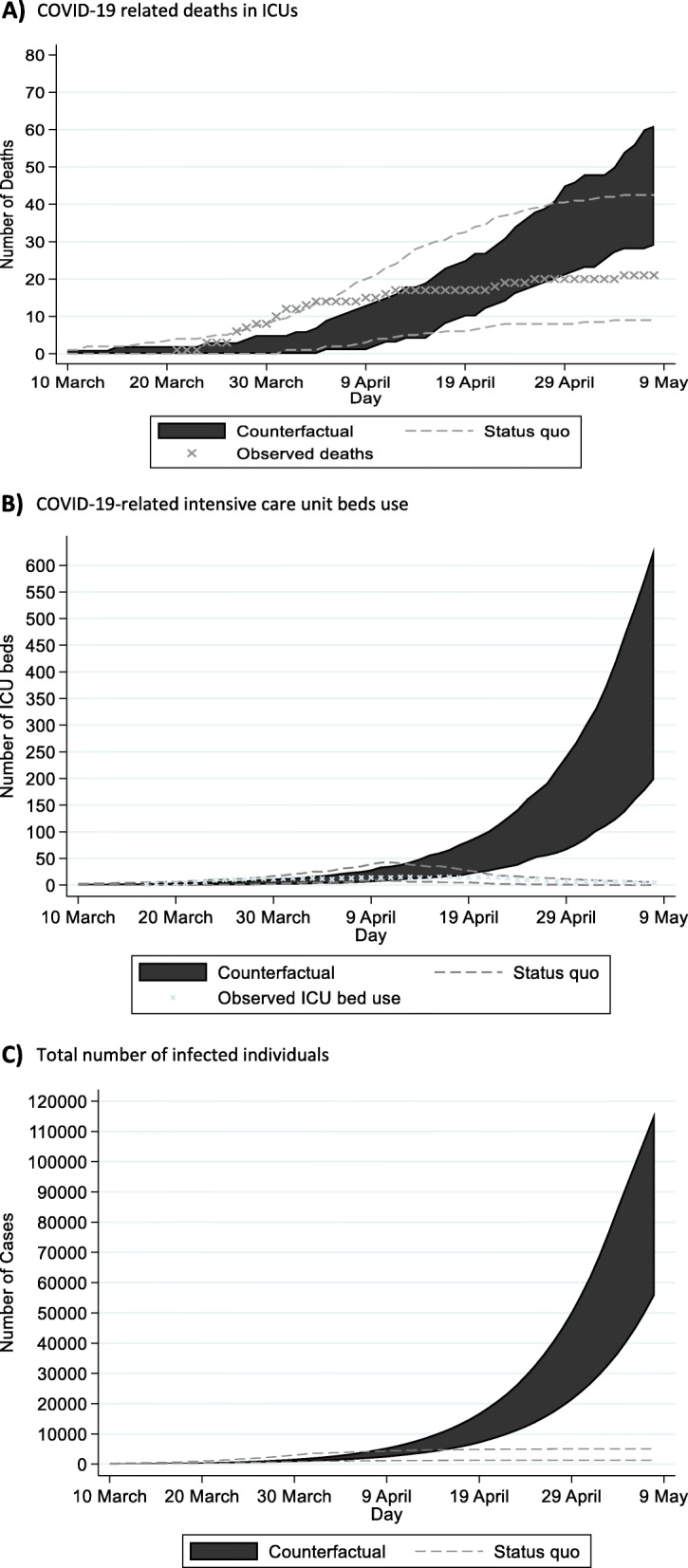
Projections of future COVID-19 cases and complications under status quo and counterfactual scenarios. For comparison, x indicates the observed trends under the status quo scenario: (A) Cumulative COVID-19 related deaths in ICUs; (B) Daily COVID-19-related intensive care unit (ICU) beds use; and (C) Total number of infected individuals

Τhe model predicted that, under the counterfactual scenario (neither social distancing measures nor increase in ICU bed capacity implemented), the total number of infected would be 86,600 (95% CrI: 46,700, 122,100) (Fig. 2C). Additionally, the pressure on the healthcare system would have been substantially higher without NPIs. More specifically, if the Republic of Cyprus had not taken any measure, the need for ICU beds would have exceeded the healthcare system capacity by April 14th (Supplementary, page 8, Fig. S6).

Under the counterfactual scenario, the projected COVID-19-related number of deaths in ICUs would be 35 (95% CrI: 23, 49) by May 8th, 2020(Fig. 2A). Considering that additional deaths could occur due to non-availability of ICU beds, COVID-19-related mortality would be significantly higher. Specifically, assuming that 95% of those in need of an ICU bed would die in the absence of available ICU beds [23], the additional deaths by May 8th would be 680 (95% CrI: 320, 1180) (Fig. 2B). Thus, the expected number of deaths without any interventions (neither social distancing measures nor increase in ICU bed capacity) would be 715 (95% CrI: 339, 1235) by May 8th, 2020.

### ICU-only scenario

If only ICU beds had increased from 27 to 50, the healthcare system would have lasted only 5 days longer from being overwhelmed, compared to the counterfactual scenario (Fig. S7).

Under this scenario, the computed COVID-19-related number of deaths would be 58 (95% CrI: 40, 75) by May 8th (Fig. S8a) and the estimated deaths due to the non-availability of an ICU bed would be 615 (95% CrI: 270, 1080) (Fig. S8b). Thus, if only ICU bed capacity had improved, the expected number of deaths would be 673 (95% CrI: 310, 1160) by May 8th, 2020.

## Discussion

Mathematical models can help understand SARS-CoV-2 transmission and assess the efficacy of measures to mitigate viral spread. Our model highlights that the non-pharmaceutical interventions (both social distancing measures and increase in ICU bed capacity) were highly successful in the Republic of Cyprus – an island state of the European Union without land borders but a popular tourist destination – as they kept the number of COVID-19-related deaths at low levels and the need for ICU beds within the capacity of the local health-care system.

The IFR is a critical indicator for the evaluation of the consequences of the COVID-19 pandemic. A recent modeling study on different settings estimated an IFR ranging from 0.5 to 1.4% [24]. Compared to those estimations, Cyprus experienced a low IFR during the first pandemic wave (central estimation: 0.71%). Possible explanations for the country’s low IFR include a) timely public health interventions, since NPIs launched before the first death, b) opportunity to improve the preparedness of the country’s health-care system, since Cyprus was not among the first affected countries, and c) the geographic position of Cyprus, which is a peripheral country of the European Union and thus less vulnerable to the effect of external seeds.

According to our estimates, only a relatively small proportion of people in the Republic of Cyprus have been infected during the first epidemic wave (IAR: 0.31% (95% CrI: 0.15, 0.54%), which is among the lowest in Europe [3]. This means that, at the end of the first wave, Cyprus was far from achieving collective immunity (the collective immunity threshold for Cyprus was estimated at 1-(1/R_0_) =1-(1/2.66) =62.4%). Under the counterfactual scenario, which resembles a strategy without viral suppression based on herd immunity from natural infections, 86,600 individuals (95% CrI: 46,700, 122,100) would have been infected, which corresponds to a prevalence of 9.9% (95% CrI: 5.3, 13.9%), still well below the target of 62.4%. Therefore, collective immunity could only be achieved in Cyprus through a combination of vaccination and infection, although the duration of immunity following infection remains unknown [25].

Globally, COVID-19 true infections are more than the laboratory-confirmed cases [3, 22]. However, case ascertainment in the Republic of Cyprus during the first epidemic wave was relatively high (33.2%). This means that, during the first epidemic wave, each individual diagnosed with COVID-19 in Cyprus corresponded to about two undiagnosed cases. The corresponding ratios for Germany, Spain, Sweden, and Greece were 23.5, 9.8, 6.1, and 24%, respectively [3, 6, 26]. It is important that the efficacy of the testing strategy to control COVID-19 is strongly dependent on the efficiency of backward contact tracing and adherence of the infected individuals to isolation.

According to our model estimates, the implemented public health measures averted 715 (95% CrI: 339, 1235) deaths. Comparing the different interventions, social distancing measures were more important to flattening the epidemic peak and reducing the pressure on the health care system than the increase of the ICU bed capacity only. Consistent with previous work [6, 7], our results highlighted that any intervention to boost only ICU bed capacity would not have been an effective healthcare policy, as the demand for ICU beds would rapidly outrun availability. Figure 2B displays that after April 10th, 2020, the demand for ICU beds would have increased exponentially.

Several studies have underlined the value of the NPIs to limit the circulation of the virus during the first COVID-19 wave [2, 3, 27–29]. Despite the efficacy of NPIs, however, it is important to take into consideration their indirect negative effects, including reductions in economic activities, mental issues due to isolation, and difficulties in accessing health care for chronic and other diseases [30, 31]. In particular, the behavioral fatigue associated with adherence to COVID-19 restrictions (the pandemic fatigue) has been a vital issue that affects the response to the pandemic, since long-period strict lockdowns may face a reduction in their effectiveness [31]. Pandemic fatigue appeared in the Republic of Cyprus too, after the end of the first wave, and increased gradually in all the subsequent waves, although the NPIs were lifted for some time. Understanding pandemic fatigue is challenging since it has several causes [32, 33]. Future work that examines the factors that drive compliance and identify those that could be modifiable are needed. Furthermore, research that seeks to identify the optimal duration of NPIs policies will be useful. All these data could inform targeted, tailored, and effective policies, interventions, and communications.

### Study implications

Our study contributes to the discussion regarding the effectiveness of NPIs during the first epidemic wave. Our results provide theoretical support that fast and accurate interventions minimized the first COVID-19 wave and prevented the overload of the healthcare system. These findings could guide both the confrontation of next waves of COVID-19 and policies during the interim period between epidemic waves when social distancing restrictions are lifted. Additionally, in periods when social distancing measures are lifted, improved surveillance and active contact tracing are very important since they are capable of timely detecting any COVID-19 outbreak very quickly. Finally, our results could be useful in designing policies in the case of an emergence of a novel disease without effective treatment or vaccines that could mimic SARS-CoV-2 transmission.

### Comparison with other studies

Our results are consistent with previous modeling studies that examined the first COVID-19 wave in the Republic of Cyprus showing that NPIs managed to limit the burden of the first wave of COVID-19 [8, 9]. However, there are two main differences between these studies and the present work. The first is that our study used the trajectories of COVID-19-related deaths, hospitalized cases, and ICU bed use as calibration points, while the other two studies used the number of reported cases. The second difference lies in the nature of the models. More specifically, in our analysis, we have used a stochastic, individual-based mathematical model while the other two studies have used compartmental mathematical models. Overall, however, all three studies underline the significance of NPIs to minimize the burden of COVID-19.

### Limitations

As with any modelling study, there are also limitations. First, the model ignores the impact of social networks in the population and assumes that it is randomly mixed. Second, an important assumption is that all deaths due to SARS-CoV-2 infection have been identified and reported (e.g., no deaths under the status quo scenario occurred before admission to the health-care facility). Third, we assumed that post-infection immunity completely protects again reinfection over the duration of simulations. Fourth, we assumed that all deaths occurred in the ICU. Notwithstanding, the effect of this assumption on our projections is likely to be marginal, since the simple-bed mortality is relatively low and no deaths outside the hospital system have been reported [5]. The above was supported also by analyses from Euromomo that showed that overall mortality in Cyprus remained within the expected range [34]. Fifth, due to the relatively low number of COVID-19 related deaths during the first epidemic wave, the model does not capture the initial numbers of deaths very precisely. Finally, in the counterfactual scenario, public health interventions were removed, while assuming that everything else remained exactly as in the status quo scenario, i.e., there would be no changes in the duration that a patient stays in an ICU bed or in hospital.

## Conclusions

Non-pharmaceutical interventions, including increased testing rates and active contact tracing, limited the burden of the first wave of COVID-19 in the Republic of Cyprus and prevented the health-care system from becoming overwhelmed. Furthermore, our results highlight that collective (“herd”) immunity could not have been achieved in the Republic of Cyprus during the first wave, even if social distancing measures were not taken. The findings of our study could be used as a guide in the confrontation of next waves of SARS-CoV-2 or of other pathogens with common routes of transmission in the Republic of Cyprus or in other similar settings.

## Data Availability

The datasets generated and/or analyzed during the current study are available in the Epidemiological Surveillance Unit of the Ministry of Health of Cyprus. National Situation Report. Coronavirus Disease 2019 (COVID-19) 2020 [updated 25/09/2020; cited 2020 14/10]. Available from: https://www.pio.gov.cy/coronavirus/pdf/ep1205ien.pdf.
